# Influence of Family Involvement and Children’s Socioemotional Development on the Learning Outcomes of Chilean Students

**DOI:** 10.3389/fpsyg.2019.00335

**Published:** 2019-02-28

**Authors:** Mahia Saracostti, Laura Lara, Diana Martella, Horacio Miranda, Edgardo Daniel Miranda-Zapata, Tal Reininger

**Affiliations:** ^1^Centro de Investigación sobre Procesos Socioeducativos, Familias y Comunidades, Núcleo Científico Tecnológico en Ciencias Sociales y Humanidades, Universidad de la Frontera, Temuco, Chile; ^2^Carrera de Psicología, Facultad de Ciencias Sociales y Humanidades, Universidad Autónoma de Chile, Talca, Chile; ^3^Carrera de Psicología, Facultad de Ciencias Sociales y Humanidades, Universidad Autónoma de Chile, Santiago, Chile; ^4^Núcleo Científico Tecnológico en Ciencias Sociales y Humanidades, Universidad de la Frontera, Temuco, Chile; ^5^Núcleo Científico Tecnológico en Ciencias Sociales y Humanidades, Universidad de la Frontera, Temuco, Chile; ^6^Escuela Escuela de Ciencias Sociales, Facultad de Educación y Ciencias Sociales, Universidad Andres Bello, Santiago, Chile

**Keywords:** family involvement, children’s socioemotional development, learning, family and school relation, child development

## Abstract

There is an extensive body of evidence to support both family involvement and students’ socioemotional development as key factors in the promotion of learning outcomes. However, there is insufficient evidence to establish exactly what this impact is when both factors are considered simultaneously. Therefore, the aim of this study is to analyze the influence of family involvement and socioemotional development on learning outcomes of Chilean students, identifying the structure that most correctly identifies the influence of the predictor variables (family involvement and socioemotional development) on learning outcomes. We present the following three hypotheses that consider possible basic interrelation structures: (1) The influence of family involvement on learning outcomes is mediated by students’ socioemotional development (mediation hypothesis); (2) The influence of family involvement on learning outcomes is moderated by students’ socioemotional development (moderation hypothesis); (3) Family involvement and students’ socio emotional development directly affect learning outcomes (covariance hypothesis). The structures were evaluated by means of a structural equation model analysis. The study included 768 students who attended second and third elementary grades in Chilean schools. The children were between 7 and 11 years old (*M* = 8.29, *SD* = 0.86); 41.3% were girls and 58.7% were boys. The results show that family involvement and students’ emotional development directly affect learning outcomes (CFI = 0.995, TLI = 0.993, RMSEA = 0.016). From the results, we can conclude that the data support the hypothesis that both family involvement and socioemotional development are predictors of learning outcomes, thereby rejecting that the impact of family involvement on learning outcomes is mediated or moderated by socioemotional development.

## Introduction

The concept of family involvement has consistently emphasized the importance of and need for family support in children’s education (Baker et al., 2016), which allows for a broader conceptualization of the roles of families and schools, their relations and the impact on children’s development (Christenson and Sheridan, 2001; Patrikakou et al., 2005; Christenson and Reschly, 2010; Yamauchi et al., 2017). *Family involvement* is understood as the family’s willingness to become involved with the school and their children’s learning, including behavioral and verbal practices in the home and school activities (Anderson-Butcher, 2006).

Several studies indicate that *family involvement* has a positive influence on *children’s socioemotional development.* (Gutman and Midgley, 2000; Fan and Chen, 2001; Garbacz et al., 2017). Several studies also indicate that *family involvement* has a positive influence on the development of children’s abilities (Epstein and Sander, 2000; Vélez, 2009; Chavkin, 2017) and particularly on school learning outcomes (Brody et al., 1999; McWayne et al., 2004). Most of the studies in this area come from Anglo-Saxon (Garbacz et al., 2017) while in the Latin American context research is still scarce.

In his meta-analysis of 51 studies into family involvement programs, Jeynes (2012) concluded that reading programs shared between parents and children, programs focused on effective alliances, and programs focused on improving the communication between home and school had the greatest impact on children’s academic performance. Another meta-analysis, after analyzing 46 studies identified that the key aspects explaining the positive correlation between learning outcomes and parental participation were the school-home connection (Ma et al., 2016). Finally, in a meta-analysis of 37 studies, Castro et al. (2015) found that the type of family participation that most affected students’ academic performance was the parents having high expectations of their children, developing and maintaining a fluid communication about what happened at school, and promoting the development of reading habits.

In addition to the literature on the positive impact of family involvement on *children’s socioemotional development and learning outcomes*, there is ample evidence of the relation between *children’s socioemotional development* and learning outcomes (Jiménez and López-Zafra, 2009). It has generally been observed that positive emotions (e.g., pleasure in learning) are positively related to academic success, whereas negative emotions (e.g., anxiety) have an inverse relation (Goetz and Hall, 2013; Pekrun and Linnenbrink-Garcia, 2014). There are also studies that have found a statistically significant relationship between Emotion Quotient Inventory EQi- scores and academic performance (Bar-On, 1997; Parker et al., 2004) crucial for the education-learning process (Humphrey et al., 2007; Pekrun et al., 2017), nevertheless the results of some studies that have analyzed the relation between academic success and socio-emotional competence present ambiguous results (Newsome et al., 2000).

**FIGURE 1:**
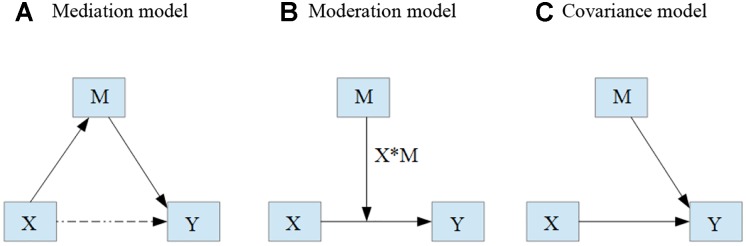
Diagrams depicting possible relations between family involvement and children’s socioemotional development on learning outcomes. **(A)** Mediation model. **(B)** Moderation model. **(C)** Covariance model. X, family involvement; M, children’s socioemotional development; Y, learning outcomes.

Due to the findings reviewed previously, we may conclude that there is evidence to support both family involvement and students’ socioemotional development as key factors in the promotion of learning outcomes. We may also conclude that there is insufficient evidence to establish exactly what this impact is when both factors are considered simultaneously. Therefore, the aim of this study was to analyze the influence of family involvement and socioemotional development on learning outcomes of Chilean students, identifying the structure that most correctly identifies the influence of the predictor variables (family involvement and socioemotional development) on learning outcomes. The following three hypotheses were considered possible basic interrelation structures (Figure 1):

(A) The influence of family involvement on learning outcomes is mediated by students’ socioemotional development (mediation hypothesis);
(B) The influence of family involvement on learning outcomes is moderated by students’ socioemotional development (moderation hypothesis);
(C) Family involvement and students’ socio emotional development directly affect learning outcomes (covariance hypothesis)

## Materials and Methods

### Participants

The study included 768 students who attended second and third elementary grades in 18 schools in regions in Chile (O’Higgins, El Maule and La Araucanía). The children were between 7 and 11 years old (*M* = 8.29, *SD* = 0.86); 41.3 and 58.7% were girls and boys, respectively.

Participants were selected using non-probability purposive sampling (Kerlinger and Lee, 2002), as is the case of the students included in this study, who come from schools that offer elementary education and have been described as having high levels of vulnerability according to the student vulnerability index issued by the Chilean Ministry of Education.

This study was carried out in accordance with the recommendations of the Chilean National Commission for Scientific and Technological Research. The protocol was approved by the Ethics Committee of the Universidad de La Frontera (Acta 066-2017, Folio 036-17). All the subjects provided written informed consent in accordance with the Declaration of Helsinki

### Instruments

#### Assessment of Family Involvement

Three scales of the Hoover-Dempsey and Sandler (2005) Parental Involvement Scale translated into Spanish and validated by a panel of experts in Chile (Reininger, 2014) were used in this study: The Parental Involvement forms (with two subscales: home based involvement, 5 items, and school based involvement, 5 items); the teacher invitations for involvement scale (6 items); and the general school invitations scale (6 items). The fist scale has a four-point Likert response scale, from 1 (never) to 4 (always), while the rest was a 5-point scale Likert response, from 1 (strongly disagree) to 5 (strongly agree).

#### Evaluation of Learning Outcomes

Three subtest of the educational psychology battery EVALÚA (García and González, 1999; García et al., 2006) were used in this study: Reading Comprehension (22 items) and two subtest of reasoning, Analogical Thinking (20 items) and Perceptual Organization (34 items).

#### Assessment of Socioemotional Development

Three dimensions of the EQ-I: YV questionnaire (Bar-On and Parker, 2000) adapted and validated in Spanish (Ferrándiz et al., 2012) were used in this study: interpersonal (12 items), adaptability (10 items) and general mood (14 items). The response scale ranged from 1 (rarely) to 4 (nearly always).

The coefficient omega average of MacDonald to ordinal scales was 0.96 to Socio-Emotional (0.78–0.97), 0.88 to Family involvement (0.78–0.84) and alpha 0.81 to Learning.

### Procedure

This study is part of a wider project focusing on the effectiveness of interventions to strengthen the link between families and schools.

The data referring to the students (evaluation of learning outcomes and assessment of socioemotional development) was collected during school hours and were registered in digital format in the schools’ computer rooms during three sessions. The data referring to the families (family involvement) were collected in paper format during parent teacher meetings.

### Analysis Plan

**FIGURE 2:**
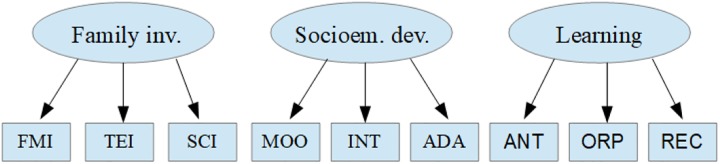
Constructs and subdimensions. Family inv., components of family involvement; FMI, father/mother involvement; TEI, teacher involvement; SCI, school involvement; Socioem. dev., components of socio-emotional development; MOO, mood; INT, interpersonal; ADA, adaptability; Learning, components of learning; ANT, analogical thinking; ORP, organizational perspectives; REC, reading comprehension.

In the first phase of data processing, inverse items were recoded, response rates were verified and corrected, unanswered records were identified and eliminated and non-parametric multiple imputation of classification and regression trees with random-forest resampling was used for missing data (Stekhoven and Bühlmann, 2012), which enables imputation for ordinal variables. In order to center the focus of the causal model on the predictive structure of the factors, the factorial scores of the subdimensions for the family involvement scale and those of socioemotional development and school learning were calculated (Figure 2), these consisting of the subscales with loads greater than 0.40 (Stevens, 2009; Brown, 2015).

Once the factorial scores of the selected subscales were calculated, these subsequently occupied the role of indicator variables for each construct. Using this configuration of components, several structural equation models were fit to determine the structure of the causal relationship between the two factors considered as antecedents (family involvement and socioemotional development) of school learning. The structures of the trajectories studied were a relation of mediation, moderation and covariance as shown in Figure 1.

In order to evaluate the fit of the models to the data, the following indices were used: comparative fit index (CFI), Tucker-Lewis index (TLI) and root mean square error of approximation (RMSEA); for the first indices, CFI and TLI, values above 0.90 or 0.95 are considered an adequate fit of the model (Schreiber et al., 2006), while for the RMSEA values below 0.08 are considered a reasonable fit (Hooper et al., 2008).

For the case of the mediation effect, statistical significance was used and the estimation of the confidence intervals by means of resampling of the specific indirect effect attributable to the presence of the mediator variable (Muthén and Asparouhov, 2015). In order to evaluate the moderation effect, a multiplicative model was used that included the product of the indicator variables of the two factors used as antecedents (Marsh et al., 2004). Finally, to evaluate the presence of a structure with covariance effect, an analysis was performed of the statistical significance of regression slopes applied to the trajectories of the latent variables of the model. The statistics software used was Mplus 7.11 as well as the miss Forest, laavan and sem Tools packages in R.

## Results

**FIGURE 3:**
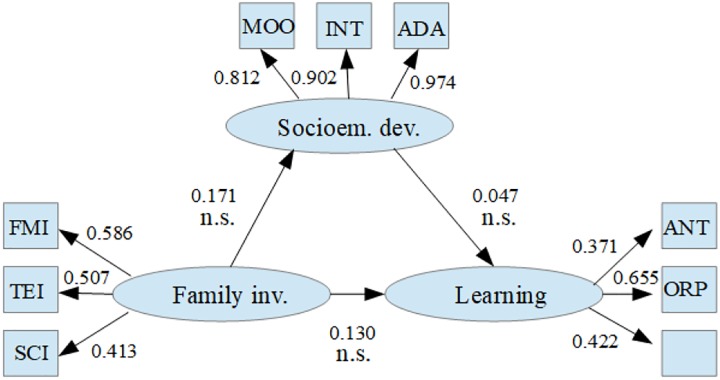
Path diagram of the theoretical mediation model. Family inv., components of family involvement; FMI, father/mother involvement; TEI, teacher involvement; SCI, school involvement; Socioem. dev., components of socio-emotional development; MOO, mood; INT, interpersonal; ADA, adaptability; Learning, components of learning; ANT, analogical thinking; ORP, organization perspectives; REC, reading comprehension; n.s., non-significative difference.

The mediation model (Figure 3) revealed low levels of indirect relation of family involvement mediated by socioemotional development to the response of school learning; these results allow the presence of an indirect and mediating effect to be ruled out, leaving open the possibility that the effect of family involvement on learning can take the form of a moderated relation or interaction, or assume a direct and independent role of socioemotional development.

The results of the model demonstrated high levels of goodness of fit, with CFI and TL values of 0.995 and 0.993, respectively; in turn, a low error of estimation was observed with a RMSEA of 0.016, which confirms the stability of the results. On the other hand, the estimations of the parameters of the mediation model showed low and non-significant levels of the mediation effect, which leads to the conclusion that mediation is not the structure of relation between the study variables (Table 1).

**Table 1:** Estimations of the structural parameters of the mediation model.

		Effect of.	Est.no std.	S.E.	Est./S.E.	Sig. Est.Std.	Sig.
Learning	<- Family inv.	0.130	0.085	1.523	0.128	0.132	n.s.
Learning	<- Socioem. dev.	0.047	0.038	1.225	0.221	0.092	n.s.
Socioem. dev.	<- Family inv.	0.171	0.113	1.513	0.130	0.088	n.s.


*Family inv., family involvement; Socioem. dev., socioemotional development; Learning, learning; n.s., non-significative difference at 5%.*

This was also corroborated by the estimation of the confidence intervals for significance levels of 1 and 5%, both for the sum of the mediation effect and for the estimation of its specific effect (Table 2).

**Table 2:** Confidence intervals for the estimations of the structural parameters of the mediation model.

Confidence interval		inf.99%	inf.95%	inf.90%	Estim.	sup.90%	sup.95%	sup.99%
Learning	<- Family inv.	-0.023	-0.011	0.027	0.130	0.319	0.366	0.460
Learning	<-Socioem. dev.	-0.020	-0.008	-0.002	0.047	0.124	0.141	0.179
Socioem. dev.	<- Family inv.	-0.125	-0.034	0.004	0.171	0.373	0.413	0.497
Ef.ind.specific		-0.013	-0.008	-0.005	0.008	0.021	0.024	0.029


*Family inv., family involvement; Socioem. dev., socioemotional development; Learning, learning; Ef.ind.specific, specific indirect effect*.

Based on the results of the evaluation of the mediation effect, it is possible to conclude that there is no significant evidence in any of the parameters of the model (*p* > 0.05) to corroborate that the relation between the study variables is a model with mediation effect. Therefore, with the hypothesis of the mediation effect being discarded, it becomes necessary to evaluate the models corresponding to the effects of moderation and covariance.

**FIGURE 4:**
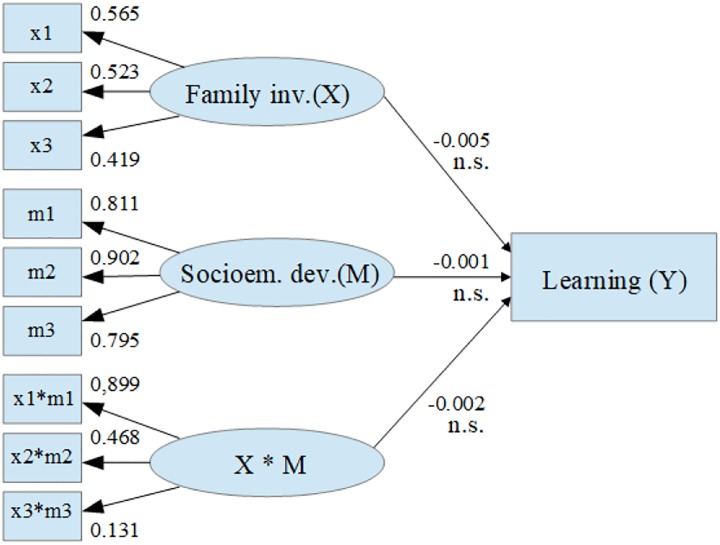
Path diagram of the moderation model. Family inv.(X), components of family involvement; x1, father/mother involvement; x2, teacher involvement; x3, school involvement; Socioem. dev.(M), components of socioemotional development; m1, mood; m2, interpersonal; m3, adaptability; Learning (Y), learning; X^∗^M, multiply of indicators variables; x1m1, father/mother involvement by mood multiply; x2m2, teacher involvement by interpersonal multiply; x3m3, school involvement by adaptability multiply; n.s., non-significative difference at 5%.

In relation to the moderation hypothesis, the results showed that incorporating the interaction effect of the exogenous variables means that all the effects are statistically non-significant (Figure 4).

This is confirmed through an analysis of the slopes of each level of socioemotional development, which are not statistically different from zero (Table 3). The intercept analysis was dismissed, as the indicator variables were centered prior to the statistical analysis of the moderation model.

**Table 3:** Regression slopes for the moderation model.

Socioem. dev.	Mod	Slope	SE	Wald	Sig.
Low	-1	-0.0024	0.021	-0.12	0.91
Medium	0	-0.0049	0.014	-0.36	0.72
High	1	-0.0074	0.021	-0.36	0.72


*Socioem. dev., socioemotional development; Mod, moderator variable level (socioemotional development)*.

**FIGURE 5:**
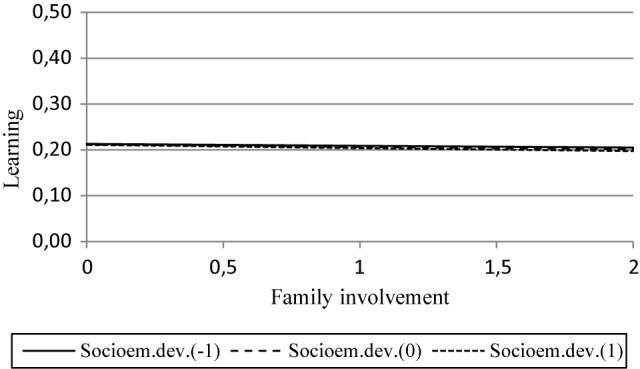
Graph of regression lines for the moderation model. Socioem. dev., socioemotional development.

These findings were also corroborated by the presence of parallelism in the graph of the regression lines (Figure 5), resulting from the interaction between the independent variable of family involvement and the variable that acted as moderator in the model, which in this case was socioemotional development.

Based on the evidence verified in the evaluation of the interaction model, it is possible to conclude that the hypothesis regarding a moderation effect of socioemotional development on the relation of family involvement and school learning is rejected.

**FIGURE 6:**
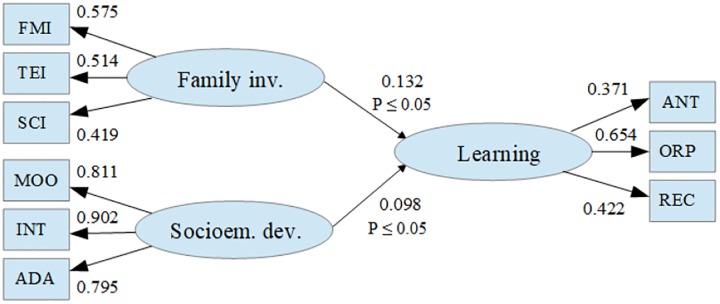
Path diagram of the covariance model. Family inv., components of family involvement; FMI, father/mother involvement; TEI, teacher involvement; SCI, school involvement; Socioem. dev., components of socio-emotional development; MOO, mood; INT, interpersonal; ADA, adaptability; Learning, components of learning; ANT, analogical thinking; ORP, organization perspectives; REC, reading comprehension. *P* ≤ 0.05, significative difference at 5%.

Finally, due to the rejection of the two previous hypotheses corresponding to the mediation and moderation models, in this section the results obtained in the verification of the statistical significance of a covariance model are described. The results showed that family involvement and students’ emotional development directly affect learning outcomes (CFI = 0.995, TLI = 0.993, RMSEA = 0.016), explaining 69% of the learning variance. This allows to conclude that in the context of the analyzed data, socioemotional development participates directly and independently in school learning in a similar way although to a lesser degree (γ_21_ = 0.098, *p* = 0.049) than family involvement (γ_23_ = 0.132, *p* = 0.032) (Figure 6).

## Discussion

International literature indicates that the degree of family involvement in school processes is a critical element in the development and learning outcomes of children during their first school years (Hoover-Dempsey and Sandler, 1997; Caspe et al., 2006) making relevant the need to generate scientific evidence from the Chilean context for eventual future replications in other Latin American countries.

The results of the present study support the hypothesis that both family involvement and socioemotional development are predictors of learning outcomes, thereby rejecting the notion that the impact of family involvement on learning outcomes is mediated or moderated by socioemotional development. In this sense, both factors are positioned as dimensions with a direct effect on learning outcomes in the case of Chilean students.

One of the main contribution of this study is its focus on Latin America due to the lack of literature from this region. For example, in a recent systematic review, only one Mexican study from 1998 appeared, which was strongly influenced by U.S. interventions (Eichin and Volante, 2018). In this context, Chile has acknowledged the importance of collaborative relationships between families and schools leading to the development a National Policy for Father, Mother and Legal Guardian Participation. Nevertheless, the majority of research in the country has thus far been of a qualitative nature with a focus on describing family school relations and identifying tensions between these two spheres (Gubbins, 2011). Thus, this study aimed to make progress in the analysis of the effect of parental involvement in school and children’s socioemotional development on learning outcomes of Chilean students.

One of the main weaknesses is that the study utilized a thematic or convenience sample. Therefore, one of the main challenges for future research in Chile and Latin America is the need for studies with probabilistic samples.

## Author Contributions

MS developed the study concept and the study design. LL and DM substantially contributed to the study concept and the study design. DM, TR, and LL performed the data collection. HM and EM-Z performed the data analysis and interpretation under the supervision of MS and LL. MS, LL, and DM drafted the manuscript. DM, TR, LL, HM, and EM-Z substantially contributed to the interpretation of the data and provided important critical revisions. All authors approved the final version of the manuscript and also agreed to be accountable for all aspects of the work.

## Conflict of Interest Statement

The authors declare that the research was conducted in the absence of any commercial or financial relationships that could be construed as a potential conflict of interest.
